# Attitudes towards data access and sharing health data for research: a case study of Australian data custodian perspectives

**DOI:** 10.1177/18333583251329533

**Published:** 2025-05-03

**Authors:** Richard J Varhol, Suzanne Robinson, Crystal Man Ying Lee, Sean Randall, James H Boyd

**Affiliations:** 1 Curtin University, Bentley, WA, Australia; 2 Deakin University, Geelong, VIC, Australia; 3 La Trobe University, Melbourne, VIC, Australia

**Keywords:** data sharing, data governance, health data, privacy, secondary use, electronic medical records, governance, stakeholders, research, health information management

## Abstract

**Background:**

The effective sharing of health data is critical for advancing public health research and improving health outcomes. Data custodians responsible for managing these data encounter various obstacles that prevent the efficient exchange of this information for research purposes. This study explores specific challenges to effective data custodianship and how they can be addressed to improve data sharing within the Australian health and human services sectors.

**Objectives:**

A case study to explore the complex landscape of data sharing from the perspective of data custodians. This includes identifying legislative complexities and related organisational cultural challenges associated with data requests that accompany the role of a data custodian and suggesting strategies to facilitate data sharing.

**Method:**

Utilising qualitative thematic analysis, semi-structured interviews were conducted to collect detailed perspectives on the practices and challenges of sharing health data from 11 data custodians across nine Australian healthcare organisations.

**Results:**

The study highlighted several common challenges affecting data sharing for research, including legislative hurdles, lack of uniform standards for data access, and inconsistent consent protocols across datasets. Internal organisational factors, such as data request assessment processes, organisational culture, and attitudes towards data sharing, emerged as critical barriers to the efficient sharing of data.

**Conclusion:**

Overcoming data-sharing barriers necessitates a multifaceted approach, requiring clear and consistent legislative frameworks for data access and establishing standards for transparent and efficient request assessment processes.

**Implications for health information management:**

Shifting data from a liability to a valuable asset can enhance decision-making, foster collaboration, and drive health sector innovation.

## Introduction

The growth of health sector data offers an opportunity to advance research and policy development. Access to comprehensive, high-quality health datasets is essential for driving innovation, informing, evidence-based decision-making and advancing knowledge (Farinelli et al., 2015; Vassiliou et al., 2020; Hollingworth et al., 2021). However, the challenges of data sharing in healthcare are multifaceted, particularly in countries with decentralised healthcare systems. In Canada, inconsistent laws, standards and formats across provinces hinder data interoperability, while conflicting governance requirements from ethics and privacy committees further complicate the process. To overcome these obstacles, healthcare systems must prioritise standardisation, centralised governance and investment in interoperable systems (Ballantyne and Style, 2017; Correll et al., 2021; Deloitte, 2015; Lumos, 2021; New Zealand Government: Government Chief Data Steward, 2022; New Zealand Government: Ministry of Health, 2021; Protti and Bowden, 2010; Protti et al., 2009). Additionally, comprehensive data governance frameworks and collaborative networks are essential for ensuring responsible and ethical data sharing. By addressing these challenges, healthcare organisations can harness the power of data-driven insights to improve patient care and outcomes.

Australia faces similar difficulties due to its federated structure, which divides responsibility between federal and state governments (Murphy and Arban, 2021). As a result, Australian researchers face many challenges when requesting data from risk-averse data custodians, whose decision-making is shaped by organisational cultures, governance structures and legislative boundaries (Katz et al., 2018). Understanding the roles and responsibilities of data stewards (who are legally accountable for the data collection) and data custodians (who administer routine tasks in accordance with the steward’s direction) (Hodges et al., 2020) in each region (see Appendix 1) becomes essential to developing a comprehensive understanding of data access processes.

Trusted research environments (TREs) have emerged as a potential solution to some of these data-sharing challenges, offering secure virtual spaces where data can be analysed while remaining under the control of the original custodians (Graham et al., 2023; Kavianpour et al., 2022; Lifebit, 2023; Torabi et al., 2023). Several initiatives are actively developing TRE-based environments (Abbasizanjani et al., 2023; Australian Research Data Commons (ARDC), 2024; Barker et al., 2019). Despite these advancements, data access is often delayed due to complex legal reviews, inconsistent data access agreements and legislative discrepancies across jurisdictions (Brophy et al., 2023; Data and Analytics Research Environments (DARE UK), 2021; Kavianpour et al., 2021).

Researchers in countries with a long history of data sharing, such as Canada (Bubela et al., 2019; Katz et al., 2018), the United Kingdom (UK) (Ford et al., 2019), and Australia (Canaway et al., 2019; Hollingworth et al., 2021; Mitchell et al., 2015; Palamuthusingam et al., 2019), have expressed common concerns with the laborious and time-consuming process of acquiring data. These barriers often delay research progress and impede scientific discovery (Bohensky et al., 2010; Mitchell et al., 2015; Palamuthusingam et al., 2019; Tenopir et al., 2011). Despite operating within distinct health systems and governance frameworks, health organisations in these countries share similar challenges in providing data for secondary use. Traditional data-sharing methods in these countries are characterised by siloed information and lengthy application processes that hinder collaboration and efficient research.

Effective management of health sector data requires careful consideration of governance structures, local legislation and organisational policies. The custodians’ roles significantly influence these factors in ensuring data-sharing compliance and governance (Hossein and MacFeely, 2023; Ugochukwu and Phillips, 2024).

This study builds on previous research (Adams et al., 2022; Allen et al., 2019) by addressing the operational and technical challenges of data sharing, focusing on legislative complexities and organisational practices faced by data custodians. Previous studies primarily concentrated on the ethical and social dimensions of data custodianship. This work highlights the need for streamlined data request processes, uniform standards for access and stronger organisational support to overcome these barriers. Practical recommendations are offered to improve data governance, suggesting centralised models both at the state and national levels can simplify the complexities of data sharing. These models can lead to more efficient data sharing and ultimately benefit healthcare research and policy development.

## Method

This study employed a retrospective qualitative interview design to explore the experiences, perceptions and perspectives of data custodians in Australia. In-depth interviews were conducted with 11 participants to gain a deeper understanding of the data-sharing processes. Semi-structured interviews allowed participants to provide detailed narratives, enabling them to share their experiences and perceptions in their own words and at their own pace. This approach facilitated the collection of richer and more contextually relevant data pertaining to the complexities, challenges and opportunities inherent in data-sharing processes from the perspective of data custodians.

### Question design

Interview questions were developed using the Setting, Perspective, Intervention/Exposure, Comparison, Evaluation (SPICE) framework (Booth et al., 2019) to guide topic exploration from a custodian’s perspective within their organisational context. While not all framework elements were directly addressed (i.e. Intervention and Comparison), the development process focussed on understanding the Setting, Participant and Evaluation of data custodianship, building on themes identified from previous Australian studies (Adams et al., 2018; Allen et al., 2019; Canaway et al., 2022; Corman et al., 2022; Flack et al., 2019; Street et al., 2021). Iterative input from the research team further refined the questions. The final set of six overarching questions, with additional probes, aimed to explore participants’ attitudes towards data sharing, focused on understanding custodian experience in fulfilling data requests, the challenges encountered during the request process and the barriers and opportunities of the role are shown in Box 1. This structured approach not only allowed for a systematic investigation of data custodians’ perspectives but also aligned with the study’s overarching goal of understanding barriers and opportunities as part of the complex healthcare landscape.

**Box 1.** Themes and Questions Used for Understanding the Role of a Data Custodian.

Theme	Question
Understanding the role	• What is your understanding of what a data custodian’s role is? [P]
	• How long have you been in the role? [P]
	• Does your organisation have a digital/data strategy? [S]
Barriers to the role	• What are some of the barriers that prevent you from doing your role [E]
Opportunities for improvement	• What does data success look like to you? [E]
	• In your opinion, how do we get people to share their data? [E]

*Note*: SPICE Elements: [S]-Setting, [P]-Participant and [E]-Evaluation.

### Recruitment

The absence of a standardised definition of a “data custodian” and the fragmented nature of the Australian health system posed a considerable challenge in identifying, engaging and recruiting a comprehensive cohort of data custodians for this study. To navigate these challenges, purposive sampling was employed to target specific types of organisations actively involved in health data collection, curation and dissemination for research purposes, including research institutes, health service providers (i.e. general practice) and organisations that collect and use data for secondary use. Data custodians were recruited from organisations with diverse primary functions and funding types across Australia, representing a variety of settings where data custodians operate.

The research team initially identified potential organisations by reviewing public records (e.g. relevant government websites and healthcare directories) and professional networks (e.g. personal experience and online communities related to health data governance), prioritising those with a recognised commitment to data governance and a significant role in healthcare data management. This review process led to selecting nine organisations relevant to the study’s objectives.

A subsequent examination of online (e.g. LinkedIn) professional profiles and organisational directories, if available, was conducted to identify individuals with roles closely aligned with health data management and governance. This search criteria focussed on keywords such as “data-sharing,” “secondary use,” “healthcare” and “custodianship” and included titles such as Program Director, Project Manager, Data Governance Specialist, Data Analyst, Health Information Manager, Professor, Senior Research Fellow and General Practitioner, which indicated a potential match with the study’s definition of a data custodian. Individuals were selected based on their involvement in data management and governance across the healthcare sector, with efforts to recruit a diverse and knowledgeable participant pool to capture a broad range of perspectives. A total of 16 individuals were identified and contacted via email, which included an overview of the study’s goals, its significance for advancing data governance practices, and an invitation to participate in semi-structured interviews. The email also outlined the ethical considerations of the study, including confidentiality and voluntary participation, to ensure informed consent.

### Data collection

Semi-structured one-on-one interviews were conducted with individuals responsible for custodianship over their organisational data. These interviews were conducted using video conferencing applications and were led by the lead author, who used an interview run sheet (Appendix 2) to guide the open-ended discussion to ensure all relevant points were covered. While focus areas were designed to guide the interviews, participants were also invited to provide additional information that they deemed relevant. Interviews were conducted over 3 months, from May to July 2022, and ranged from 30 to 50 minutes. All interviews were digitally recorded and transcribed manually by the lead author soon after to ensure an accurate conversation record.

### Analysis

The collected data were analysed using an interpretive phenomenological thematic analysis approach, which is flexible and adaptable to structured and semi-structured interview formats as used in this study (Braun and Clarke, 2006; Brinkman and Kvale, 2019; Smith et al., 2009). This approach is rooted in phenomenology, a philosophical perspective that emphasises understanding and describing the lived experiences of individuals. The transcribed interviews were analysed to identify the main themes and inductively coded using NVivo software (v1.7.1). To minimise interviewer subjectivity and topic bias, a reflexive approach was utilised by documenting personalised perspectives and potential influences on the coding process. Despite the structured nature of some of the interview questions, the open-ended section within the interview allowed for deeper exploration into the participants’ personal experiences, as encouraged by the phenomenological methods. By focusing on the lived experiences of participants, adhering to a systematic analysis process, and promoting reflexivity, this approach ensured that the research findings were grounded in the data and faithfully represented the perspectives of those being studied. The open-ended prompts within the structured interview format allowed for detailed narratives to be collected and aligned with the phenomenological outcomes. A hierarchical coding template was developed on a subset of data and iteratively refined by the authors (RV and SR1). The generated codes were subsequently themed and applied to the transcribed data by selecting illustrative quotes from the participants. Central themes were identified and classified across the interviews and reviewed by RV and SR2 for consistency. To enhance clarity and conciseness, irrelevant text was omitted and replaced with ellipses.

### Ethics

All participants gave verbal and written informed consent after receiving the study’s information guide explaining the purpose of the interview and how their data would be securely processed, ensuring they remained anonymous. The project was governed in accordance with ethics approval from the Curtin University Human Research Ethics Committee (HRE2019-0619-18).

## Results

### Participant characteristics

**Figure 1.**
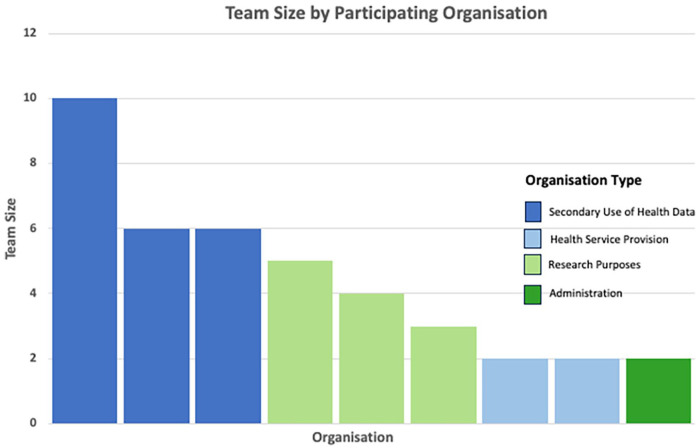
Comparison of team sizes across different participating organisation types.

Of the 16 data custodians invited to participate, 11 individuals from 9 organisations agreed to be interviewed. All participants identified as being involved in data custodianship. Participants included ten females and one male, aged 31–58 years. Although the female participation rate was high, it aligns with the broader trends in healthcare, where women make up a substantial proportion of specialised health information management and custodial responsibilities (Gjorgioski et al., 2023; Kyabaggu et al., 2022; Walsh and Borkowski, 1995). The median time in the role was 10 years, ranging from 5 to 20 years. Organisations were categorised into small (⩽5) (*N* = 7) and large (>5) (*N* = 6) groupings based on the team size (Figure 1). A team was defined as a group of individuals within an organisation including data custodians, lawyers, IT specialists and associated staff who collectively contribute to data governance related to requested data.

Organisations were grouped into four categories based on their primary function in relation to health data: (1) health service provision; (2) administrative, managing healthcare logistics and finances; (3) research purposes, which involve conducting original research using collected health data to generate new knowledge through hypothesis-driven investigations; and (4) secondary use of health data, which focuses on analysing existing datasets for purposes beyond their original intent including quality improvement and policy development (Table 1). Finally, each organisation was assigned a funding type based on the organisation type and its jurisdictional reach (national or state).

**Table 1.** Organisational representation of participants.

Team size	*N*	Organisation type	Funding type
Small (≤5)	7		
		Research purposes (*n* = 4)	• Grant funded (*n* = 3) • Mixed (Federal government and grant funding (*n* = 1)
		Health service provision (*n* = 2)	• Mixed (Federal and non-government (*n* = 1)
		Administrative *(n* = 1)	• State government *(n* = 1)
Large (>5)	4		
		Secondary use of health data (*n* = 4)	• Federal government (*n* = 3) • State government (*n* = 1)

*Note: N* = total number of participants.

Interview responses underwent coding, analysis and expansion, originating from the three primary themes and ultimately leading to the development of nine sub-themes (as outlined in Table 2). This process offered deeper insights into participants’ experiences, their readiness to engage in research, their grasp of data-sharing requirements, and associated apprehensions.

**Table 2.** Summarised themes obtained from interviews.

Main theme	Sub-theme
Role understanding	• Role and responsibilities • Capability and confidence • Organisational considerations
Barriers	• Policy and compliance • Varying data handling processes • Organisational practices • Institutional inertia
Facilitators	• Centralised data sharing models • Exemplars of good practice

Themes and exemplar quotes are included in Appendix 3 (online supplement) and discussed here.

### Understanding the role of a data custodian

Participants described their roles as data custodians, emphasising the primary duty to ensure data’s ethical use, privacy and secure management throughout its lifecycle, including tasks from collection to coding. Understanding of this role did not significantly differ across organisation types.

### Role and responsibility

The responsibility of a data custodian varied across participants. For some, it was an executive position, others identified it as a departmental duty or even a part of existing duties, often seen as an extension to IT or senior staff positions. A quote from a former NHS employee [DC03] highlighted that data custodianship could sometimes be an added responsibility rather than a defined role:

When I worked with the NHS it wasn’t really a role. It was just like a thing to do and it would sit in the IT department. It wouldn’t be a distinct role. . . it would be part of somebody’s existing job as an add-on. [DC03]

Participants also mentioned evolving into their roles, with data management responsibilities growing over time. One noted that what started as a part-time job quickly became a full commitment:

I evolved into the role because it’s certainly not my qualification at all. I came into [the role] thinking that it was a part-time job, but actually, it quickly turned into a full-time role. [DC04]

Data custodians handled data management, compliance, access control and balancing research needs. Duties varied by organisation size. Smaller non-profits often had one person handling multiple tasks, leaving limited time to focus on specialised areas such as governance, ethics and stakeholder engagement. In contrast, larger entities benefitted from specialised teams, where responsibilities were distributed, enabling a more comprehensive approach to data custodianship.

### Capability and confidence

Most participants reported a lack of formal training tailored to their data custodianship role, relying instead on experiential learning from on-the-job exposure. Some highlighted proactively searching for information due to gaps in organisational and legal knowledge and the absence of specific training programs for their specialised roles.

I’ve had to go down the path of getting the policies and procedures . . .. I’m thinking that’s not enough. [DC02]

Despite lacking formal training, custodians relied on their existing knowledge of data linkage, ethics and governance. Their ability to leverage past experience and self-directed learning varied by organisational size and resources. Larger institutions offered structured training, fostering mentorship and confidence. Smaller organisations relied on individual initiative and existing knowledge.

### Organisational considerations

Size, type and funding influenced the organisations’ data-sharing capabilities. Smaller organisations, often grant-funded, struggled with managing data sharing across multiple jurisdictions due to limited resources. Despite this, their smaller scale allowed more dynamic decision-making and agile responses to data requests, as one participant noted:

We’re not government. We can do what we need to do. . . a lot of the times it comes down to money and time and agreements and the contracts. [DC01]

In contrast, larger organisations, funded by state or federal governments, primarily managed health data for secondary use, benefitting from extensive resources and specialised teams. These organisations could efficiently manage their datasets due to their capacity to employ specialised staff dedicated to data governance, legal compliance and privacy protection. As one participant noted:

Part of the puzzle for us is actually how we manage the data and how we transform the data for it to be used,. . . we have many individuals who contribute to our governance process. . .we also have a lawyer on staff who is our privacy officer. . . we have another number of individuals who all have roles and responsibilities to play to ensure that the data are well governed. [DC07]

Although smaller organisations engaged in multiple research projects, larger organisations involved in similar collaborative research were better equipped to manage complex data requests due to their established processes and access to dedicated teams for navigating data-sharing regulations.

### Barriers

When discussing barriers, data custodians encountered diverse challenges related to legislative disparities, request processes and organisational culture. The challenges highlighted an intricate interaction of legal, procedural, cultural and organisational factors that collectively hinder data exchange.

### Policy and compliance

All organisations adhered to privacy laws, but one [DC03] exceeded the requirements in order to build trust. This proactive approach fostered support and emphasised data utility. Others faced challenges due to inconsistent legislation across jurisdictions and organisations. This variation created unnecessary complications:

The issue here is that our legislation around our data is at a State and Territory level so we have to contemplate provisions in the Health Administration Act, the Health Records and Information Privacy Act, Personal Privacy and Information Protection Act (PPIPA), and possibly in Public Health Act for certain data collections. All these require consideration when it comes to a decision whether we release our data or not. [DC06]

Participants noted legislative barriers preventing data sharing between groups, suggesting outdated legislation. Participant [DC04] advocated for a review and update to facilitate sharing and collaboration. Data custodians identified inconsistencies in consent requirements as an administrative barrier [DC02], suggesting standardised consent frameworks could improve the assessment of data requests and governance compliance. Varying levels of consent across datasets, such as differences in whether consent is broad, specific or conditional, can be restrictive, hindering research and limiting the ability to gain a full understanding of the study population.

### Data handling processes

All respondents had transparent data request processes. Requests were similar, with external researchers preparing initial applications. Internal reviews followed, with complex requests requiring expert assessment. Larger organisations integrated assessments. Participants offered quick turnarounds for simple requests; however, more complex requests, involving extensive assessments or approvals, could take up to 12 months.

Although most institutions mentioned having a single transparent data request process, only government organisations [DC07, DC02] acknowledged a separate expedited track for ministerial requests, which also required going through appropriate ethical and governance procedures.

Custodians were cautious about providing processing times, citing factors like inconsistent requests, disparate datasets and limited capacity. These factors could lead to delays.

One custodian [DC05] identified the delays researchers face when receiving requested data, while another custodian expressed similar concerns about similar delays for data processed through a State Health department, sometimes taking over 2 years [DC04].

### Organisational practices

Custodians highlighted the struggle within organisations regarding the decision to share data, often tied to concerns over trust and reputational risks. The sentiment that trust, which takes years to build [DC08], can be quickly destroyed underscores the cautious approach to data sharing.

Some mentioned being influenced and constrained by their organisation’s lack of political will or their management’s unfamiliarity with specific datasets, leading them to adopt a risk-averse stance. One participant [DC09] noted that a sense of territorialism associated with data ownership exacerbated this conservatism, adding to delays and restricting custodians from gaining the autonomy needed to make informed decisions about data access and sharing.

Organisations often grapple with risk aversion due to uncertainties surrounding data release policies, processes and procedures. This, combined with a lack of confidence, results in a focus on privacy and risk management, which often overshadows the data’s potential value.

### Institutional inertia

Political will was a barrier to data sharing in government institutions. Organisations can be possessive about their data, fearing loss of control. Clear data governance frameworks could mitigate concerns by establishing transparent rules and processes that define how data are shared, used and managed, further providing clarity on data ownership, responsibilities and accountabilities, ensuring organisations retain oversight while enabling controlled access to third parties. Additionally, these frameworks could include mechanisms for recognition and attribution of research outcomes and overcome the disincentive of future data sharing:

Those in the echelons of leadership don’t understand what data science is, and perhaps we’re still educating upwards. . . .. I think there is that element of not understanding how your data can improve things or inform better decision-making. [DC04]

### Facilitators

Participants emphasised the importance of consistent request and processing models, noting consistency, transparency, shared expertise and streamlined governance, played a role in facilitating data access. Standardised forms, automated tracking and dedicated support facilitated easier data access [DC02]. Some custodians implemented centralised data repositories and integrated workflows to improve data sharing [DC01]. To address varying request processes, one organisation invested in infrastructure and data engineering [DC01].

Participants identified transparency, consumer engagement and accessibility as key areas of interest. Australia’s National Prescribing Service’s MedicineWise (National Prescribing Service (NPS), 2022; Consumers Health Forum of Australia and NPS MedicineWise, 2018) was recognised for its rapid review process, consumer engagement and broad governance framework of primary care datasets. Similarly, the New South Wales LUMOS initiative (Correll et al., 2021; Lumos, 2021) was identified for addressing legislative barriers associated with primary care linkage, while New Zealand’s health system (Ballantyne and Style, 2017; Deloitte, 2015; Protti et al., 2009; New Zealand Government: Government Chief Data Steward, 2022; New Zealand Government: Ministry of Health, 2021) was acknowledged for government-mandated data standards and commitment to data sharing. The UK’s NHS (Anandaciva, 2023; Department of Health and Social Care, 2023) stood out for its transparent and centralised systems, with Manitoba’s Centre for Health Policy (Katz et al., 2020; Smith et al., 2018; University of Manitoba, 2024) recognised for effective data and code-sharing practices. Additionally, respondents unanimously supported consistent, standardised processes in place to garner trust and facilitate efficient access to data while ensuring adherence to privacy principles and data security principles.

## Discussion

Despite its significance for efficient healthcare delivery and research, data sharing is frequently impeded by various obstacles. Our findings highlight the multifaceted nature of the data custodian role, its recognition within organisations and its adaptive evolution.

Existing literature reveals a gap in studies on data custodians and attitudes and experiences, particularly in healthcare. Previous research has predominantly focused on governance and privacy from an organisational or policy perspective (Harron et al., 2017; Jones et al., 2019a; Smith et al., 2018). For instance, Smith et al. (2018) and Jones et al. (2019a) discussed governance frameworks, but they did not explore the individual experiences of custodians. While substantial literature covers technical and regulatory aspects (Basu and Guinchard, 2020; Scheibner et al., 2022, 2023), it often overlooks the practical challenges custodians face daily. This study addresses this gap by providing insights into the challenges and facilitators of data custodians, underscoring the need for targeted research.

Research suggests the roles and responsibilities of data custodians, those responsible for data collection and management determining its use and release for research (Adams et al., 2018, 2019), can vary across jurisdictions, as they balance data confidentiality with optimising healthcare outcomes (Jefferson et al., 2022; Kardash and Morin, 2022). Our findings support this, suggesting this variation is associated with the organisation’s size and type. In smaller, not-for-profit research-related entities, data custodians had broader responsibilities. Conversely, in larger federally funded organisations, these duties were distributed across a more comprehensive skill set where specific individuals manage specific aspects of the data requests, including jurisdictional governance requirements.

Common to all respondents was the dynamic nature of their role. In certain instances, custodianship was an additional responsibility appended to existing roles, implying that responsibilities expanded as data became increasingly pivotal within their organisations. This variation reflects the early stages of the diffusion of the innovation curve (Rogers et al., 2014) as the availability of large data assets becomes more prominent. Similar variations have been reflected across Australian States and Territories (ACT Government, 2020; Government of Western Australia, 2019; Northern Territory Health, 2018; NSW Health, 2019; Tasmanian Government, 2020; The Secretary of the Department of Treasury and Finance, 2020; Government of South Australia, 2015; Queensland Government Chief Information Office, 2016), between organisations (Australian Institute of Health and Welfare, 2021; Lumos, 2021; MedicineInsight, 2021) and identified in previous studies (Allen et al., 2019; Flack et al., 2019), highlighting the need for clarity and consistency in the expectations and responsibilities of the role. As digital transformation develops, existing laws, policies and practices must evolve to accommodate these secondary uses, necessitating a period of adaptation and amendment. This study identifies a growing need for new governance frameworks to address the complexities in data management, including issues of privacy, security and ethical use.

### Policy and compliance

In Australia, data collection is decentralised, spanning multiple administrative levels and jurisdictions, each with its own unique datasets. This introduces a layer of complexity as was reflected by our cohort of respondents who indicated legislation to be complex, ambiguous and sometimes contradictory, creating uncertainty about their legal obligations. Clear consistent legislative frameworks across jurisdictions ensuring efficient navigation of the legal requirements (Scheibner et al., 2023) are needed. Complexity does not arise from fulfilling confidentiality obligations, which are generally well understood and expected, but from the varied legislative approaches and interpretations across jurisdictions regarding how to maintain privacy for individuals and confidentiality for datasets. Time-constrained custodians with an aversion to risk may feel overwhelmed by the variation and ultimately prevent the data from being released, limiting its utility for research and policy development. Initiatives such as UK’s NHS Digital (NHS England, 2023) and New Zealand’s Ministry of Health have established central bodies to manage health information to reduce the complexities of custodianship.

### Data handling processes

Processing time varied based on factors such as request complexity, number of databases involved and client responsiveness, with expedited requests requiring a deep understanding of the research purpose and knowledge of relevant databases. Participants reported significant barriers with the data request assessment process, including time-consuming and resource-intensive activities, the complexity of requests and the challenge of balancing privacy with data access. Intricate requests can cause delays in processing and overburden resources, especially when data custodians lack sufficient experience to assess requests effectively. A lack of standardisation in request assessments and communication can lead to delays, misunderstandings, and ultimately reduced data sharing, placing many research projects in jeopardy (Bohensky et al., 2010; Palamuthusingam et al., 2019).

These findings suggest incorporating specialist roles, including data analysts or statisticians familiar with research design who, together with the custodian, can evaluate the methodological soundness of data requests. Alternatively, these skills may be democratised through a centralised online version control system, such as Git (Torvalds, 2011), which tracks changes to files, enables collaboration and maintains a history of modifications. This can facilitate collaboration, encourage standardisation and reduce the processing burden faced by custodians.

Centralised systems, including the SAIL Databank (Abbasizanjani et al., 2023; Jones et al., 2017; Jones et al., 2019b; SAIL Databank, 2023), Canadian Institute for Health Information (Lucyk et al., 2015) and Manitoba’s Population Research Data Repository (Smith et al., 2018), were developed to improve data sharing by establishing transparent request and assessment processes and standardising communication between data custodians and requestors. In Australia, the Australian Institute of Health and Welfare (AIHW) is a government organisation that promotes the management of health data through initiatives such as the Secure Remote Access Environment (SRAE) (AIHW, 2021a, b) and the National Health Data Hub (NHDH) (AIHW, 2024). The NHDH is a system designed to harmonise data governance across Australia’s federated healthcare system. By providing a single access point for integrating datasets from diverse sources, the NHDH enhances the capacity for multijurisdictional research while maintaining compliance with privacy and ethical standards. This initiative is important for overcoming challenges in accessing health data for comprehensive and policy-relevant studies. Recognising the need for similar centralised systems, some custodians mentioned actively implementing these improvements in local infrastructure and workflows.

### Organisational culture

Several data custodians recognised challenges associated with their organisations’ willingness to share data often associated with concerns over trust, reputational risks and a lack of dataset familiarity, which collectively amplified their risk-aversion. While most experienced custodians were enthusiastic about data sharing, particularly for secondary uses that they considered acceptable, management was often identified as an obstacle. Custodians well versed in their datasets and legal and governance frameworks were more likely to receive management support (Wai Fong Boh and Yellin, 2006) and succeed in their data-sharing efforts (Hanson et al., 2020; Jones et al., 2019a; Zabijakin-Chatleska and Cekikj, 2020).

Despite a stated willingness of some custodians and organisations to share data, actual sharing was often limited by various factors (Wallis et al., 2013). Leadership may not fully appreciate the data’s potential for informed decision-making (Aitken et al., 2016; Heeks, 2000; Mosconi et al., 2019) or view it as a strategic resource to be used with political intent (Martin Beno et al., 2017), especially during crises (Fidler, 2008) and the recent COVID-19 pandemic (Baxter et al., 2023; de Bienassis et al., 2022).

Our study aligns with the concept of social licence (Adams et al., 2018) by highlighting the custodian’s role in maintaining community trust through responsible data management. Similar to earlier findings (Adams et al., 2018; Adams et al., 2022; Adams et al., 2018; Allen et al., 2019;), our research supports transparency, ethical considerations and community engagement in data sharing. Custodians expressed concerns over trust and reputational risks, indicating the priority of protecting individual privacy and confidentiality. This protection is essential for securing community trust, and foundational for the ongoing use of health data in research. Additionally, our study emphasises the need for clear governance frameworks and better training to manage data-sharing complexities and to maintain a social licence.

Cautious approaches towards data-sharing (van Panhuis et al., 2014) result in inconsistencies in the approval and release procedures (Adams et al., 2018; Allen et al., 2019; Flack et al., 2019). This perception of complexity creates barriers, contributes to protracted timelines and deters researchers from making requests. Traditionally, data-rich organisations prioritise risk mitigation and view data as a liability, making it a barrier to research access. However, researchers can help shift this perspective by acting as intermediaries between organisations. By collaborating with leadership, researchers can highlight the value of the data as a neutral asset. Educational workshops and collaboration can foster an understanding of data-sharing benefits, leading to improved data access for research purposes.

### Facilitators

While respondents identified similar barriers to those of a recent Canadian study (et al., 2021), participants recognised the need for improved infrastructure and mechanisms to help foster data sharing and associated processes. These observations are in support of the recommendations put forward by Australia’s 2017 Productivity Commission Report on Data Availability and Use (Government of Australia, 2017), which calls for reforms to the country’s data system, including the establishment of a National Data Commissioner, Accredited Data Authorities, and the introduction of a Data Sharing and Release Act. Guided by centralised approaches such as the SAIL Databank, some custodians are implementing similar models through infrastructure enhancements and improved workflow processes, promoting more effective data sharing and utilisation. The advantages of these models include improved efficiency, reduced complexity in data access and enhanced trust between data providers and users. Although these solutions require significant infrastructure investments, the Trusted Research Environment (Lifebit, 2023; Data and Analytics Research Environments (DARE UK), 2021) approach developed in the United Kingdom and adapted in Australia by the ARDC, offers a centralised cost-effective alternative.

Achieving a ‘future state’ with improved data availability, transparency, integration and sharing requires addressing critical challenges. These include securing funding for the development and maintenance of infrastructure, mitigating privacy risks associated with centralised consent, and ensuring the consistent and accurate application of common identifiers across data sources.

### Limitations

The study has some limitations. First, the sample size is relatively small, and the participants were drawn from the Australian healthcare sector using purposive sampling.

These findings, therefore, may not be generalisable to other contexts or sectors. However, due to the consistent variation of data custodian roles and responsibilities across data collection organisations and jurisdictional boundaries, it is felt that the findings are both timely and relevant. Second, the study relied on self-reported data, which may be subject to response bias. To address these limitations, future studies with an expanded sample size, with the inclusion of participants from a broader range of sectors and contexts, should be considered. Additionally, future studies could employ mixed-method approaches to triangulate data and reduce potential bias. Furthermore, these findings may inform the development of centralised models for data sharing with a focus on transparency, consistency and accessibility.

## Conclusion

Our findings highlight the need for further clarity and consistency in the expectations and responsibilities of the data custodian role, as well as for harmonised legislative frameworks that reduce jurisdictional variation and improve compliance. Addressing legislative complexities, including contradictory or ambiguous laws, is essential to reducing uncertainty for data custodians and enabling more efficient and collaborative data sharing practices. Data custodians face significant barriers related to the data request assessment process, including time-consuming and resource-intensive activities, organisational constraints related to available resources, the complexity of the requests, and the challenge of balancing privacy and data access. Equally important are organisational practices, such as the presence of specialised teams, standardised request processes, and internal attitudes towards data sharing, all of which influence custodians’ ability to navigate these challenges effectively.

Variability in consent requirements across datasets is an administrative burden that can limit cohort completeness and prevent optimal use of some restricted datasets. Striking a balance between data privacy and utility is paramount to addressing consent-related challenges requiring innovative approaches with the consideration of community engagement to develop ethical models of data consent to facilitate data sharing. Additionally, organisational culture can play a significant role in determining whether data custodians are able to share data, even if they are willing to do so. Despite these challenges, there is a growing recognition of the importance of data sharing, and initiatives are underway in Australia and other countries to improve the infrastructure and mechanisms for data sharing.

## Supplemental Material

sj-docx-1-him-10.1177_18333583251329533 – Supplemental material for Attitudes towards data access and sharing health data for research: a case study of Australian data custodian perspectives Supplemental material, sj-docx-1-him-10.1177_18333583251329533 for Attitudes towards data access and sharing health data for research: a case study of Australian data custodian perspectives by Richard J Varhol, Suzanne Robinson, Crystal Man Ying Lee, Sean Randall and James H Boyd in Health Information Management Journal

sj-docx-2-him-10.1177_18333583251329533 – Supplemental material for Attitudes towards data access and sharing health data for research: a case study of Australian data custodian perspectives Supplemental material, sj-docx-2-him-10.1177_18333583251329533 for Attitudes towards data access and sharing health data for research: a case study of Australian data custodian perspectives by Richard J Varhol, Suzanne Robinson, Crystal Man Ying Lee, Sean Randall and James H Boyd in Health Information Management Journal

## Appendix

**Appendix 1.** Roles and responsibilities of data custodians and data stewards in Australia.

State/Territory	Data/Information Custodian	Data Steward
Australian Capital Territory	Accountable for data governance decisions for datasets and authorising safe data access, use and sharing (1).	Responsible for operational data management and decisions for datasets. Facilitate data access, use and sharing (1).
Northern Territory	A delegated responsibility for the ongoing development, maintenance and review of data collections and quality and provision of the data, its security, timeliness and adherence to standards.(2)	Responsible for the overall strategic direction of data collections to ensure they are developed, maintained and utilised in accordance with the strategic goals and use consistent and appropriate data standards. Data Stewards are also responsible for authorising the access, use and disclosure of data from a data collection for specifically defined purposes.(2)
New South Wales	Responsible for day-to-day management and oversight of the asset, approval of access to data and the overall quality and security of the asset.(3)	Data Stewards are responsible for the day-to-day management and operation of the data asset including its completeness and quality.(3)
Queensland	The Information Custodian is responsible for implementing and maintaining information assets according to approved rules, ensuring aspects such as quality, security and accessibility are maintained throughout the asset’s lifecycle. (4)	An “Administrator” implements the requirements for the data asset specified by the custodian with responsibilities likely to be distributed among different roles. (4)
South Australia	Involves managing data quality and ensuring the data fulfills the quality criteria for subsequent utilisation. Additionally, they are responsible for maintaining the completeness, accuracy, consistency. Ensuring the currency of business and technical metadata associated with their data collections including the validation of metadata availability ensuring its alignment with the data asset’s requirements.(5)	Data Stewards assume the responsibility of recording the specifics, guidelines and allowable values in compliance with the established standards, ensuring the development of comprehensive, transparent and instructive criteria. (5)
Tasmania	Assumes responsibility for the “ownership” of service-specific data, encompassing data integrity, validation and interpretation, while facilitating the broader strategic stance for data re-utilization and authorizing access/release in alignment with institutional policies, legislative mandates, privacy and additional guidelines.(6)	Responsible for data entry and data quality including the provision of information to investigators on datasets available to assist in the feasibility assessment for research activities requiring data requests.(6)
Victoria	Assigned the responsibility of managing information throughout its lifecycle, encompassing aspects such as transport, storage, utilization and access. (7)	Responsible for specific data and information content and context. (7)
Western Australia	Accountable for the daily administration of a data collection and entrusted with supervising the reporting and monitoring requisites for data quality and enhancement, adhering to the stipulations of associated policies.(8)	The designated role assumes responsibility for devising the strategic trajectory pertaining to data quality management and facilitating the execution of data quality enhancement strategies. (8)

*Notes*:

1. ACT Government. Data Governance and Management Policy Framework. 2020.

2. Northern Territory Health. Data release guideline. 2018 25/07/2018.

3. NSW Health. Data Governance Framework. NSW Health; 2019 14 March 2019.

4. Queensland Government Chief Information Office. Queensland Government Enterprise Architecture. Information management roles and responsibilities. 2016.

5. Government of South Australia. Data Quality Management Policy Guideline. SA Health; 2015.

6. Tasmanian Government. Research Governance Policy Framework. Hobart: Department of Health, Tasmania; 2020.

7. The Secretary of the Department of Treasury and Finance. Victorian Digital Asset Strategy. Melbourne: Department of Treasury and Finance; 2020.

8. Government of Western Australia. Data Quality Policy. Perth: WA Department of Health; 2019.
