# Supplementary material for: Attitudes towards data access and sharing health data for research: a case study of Australian data custodian perspectives
Source: Health Inf Manag. 2025 May 3;55(2):315–27. doi: 10.1177/18333583251329533 (PMC13187238; doi:10.1177/18333583251329533)
Supplement: sj-docx-1-him-10.1177_18333583251329533 – Supplemental material for Attitudes towards data access and sharing health data for research: a case study of Australian data custodian perspectives [file sj-docx-1-him-10.1177_18333583251329533.docx]

| **Main Theme** | **Sub Themes** | **Minor Themes** | **Illustrative Quotes** |
| --- | --- | --- | --- |
| Role Understanding | Role and Responsibilities | Role recognition | “When I worked with the NHS it wasn't really a role. It was just like a thing to do and it would sit in the IT department. It wouldn't be a distinct role. It probably would be more now that the privacy legislation is coming in Australia stronger in the last couple of years that these sort of things [data custodianship] will come up. I think before that if you knew about it, you might have a role, but it would be part of somebody's existing job as an add on. [DC03]”  ‘’A data custodian is effectively … a manager or in an executive role, but is responsible for – not necessarily, the day-to-day nitty-gritty of the data asset, and not necessarily for the funding associated with the data asset; but that level in between, where you're responsible for engaging with your stakeholders, establishing data standards, and any kind of policy and procedures around collection, use and disclosure, and facilitating the sharing of data for purposes where it's lawful and ethical. [DC06]”  “The Data Custodian's role is to ensure that there are transparent understanding and sufficient documentation between the authoring entity (i.e. who we're getting data from) and the data custodian (i.e. us). That gives that individual [requestor of the data] confidence as to how we manage and curate their data, and how their data are used… but remember, none of us individually are data custodians. It’s the organisation that’s the custodian and it would be extremely rare to have a single individual that had every single skill set that you need to be able to be the value trusted custodian and we can benefit from a broader team. [DC07]”  “A data custodian is an exec ownership and then somebody underneath that role would be the data steward. However, some larger organisations might have a data custodian that's in the department rather than at the executive level, because that’s how it’s structured. If you get a big health organization you won't tend to get an executive that’s directly responsible, rather you’d typically see them accountable for the data as a whole including IT and the data custodian or sit below that. [DC03]”  “I would say I evolved into the role because it’s certainly not my qualification at all. I came into [the role] thinking that it was a part-time job, but actually, it quickly turned into a full-time role because it involved establishing the governance arrangements on how to apply to [a particular project] and negotiating with each of the data custodians around establishing what the criteria are in the terms of reference and then publishing and making it available for public consumption. That's just taken quite a lot of time. [DC04]”  “I would say I've grown in that trajectory over the years, as the data has become more of a mainstream, desired data set to use. I now have more of a holistic role which is bringing all the pieces of the puzzle together and providing more of an advisory, coordinating lens to make sure that these multidisciplinary functions all come together. [DC07]” |
|  | Role and Responsibilities | Responsibilities | “I'm the data manager of the project, so I help manage all the data activities within the study, but in particular, we do have our own [project] data source, which contains all of the information about our participants. This includes questionnaires, appointment data and information that comes to us by other means such as hospital, pathology, radiology or other information. For all that information contained in the database, I would say I’m the custodian of. [DC01]”  “We collect the data then we create data dictionaries around it and try and identify limitations with the data and we're also involved with doing feasibility assessments of researchers' request to use the data. It's our responsibility also to make sure that it's delivered into a safe space and only the variables that have been approved are delivered. We also advise on feasibility and usefulness of the data [DC05]”  “…I'm also working with people who want to use the data and [get an] understanding of how they're going to navigate access to the data. I’m also applying a governance lens as well because you're always thinking – ‘is what they [requestor] want to do fit for purpose?’ I need to understand the research request. How and why do they want to use the data and whether it can happen or not? I'm also overseeing the engagement with the [stakeholders] who participate in the program as well. So, we're continually getting feedback on how [stakeholders] want to be part of the program and where the pain points are and ensuring that there is a benefit. An additional part of the puzzle for us is actually how we manage the data and how we transform the data for it to be used, and I have a role in that as well. [DC07]”  “I have quite an interesting role I have responsibility for, like the ethics coverage for those collections falls to me. So I need to monitor them and am responsibility for managing their appropriate access and the conditions of their access, the release of the data in that kind of thing …. I’m also considered the subject matter expert. I do clearances of reports that contain specific information….related to the sector or the environment, ensuring it is described and represented appropriately as well as picking up any issues with the way data have been presented within that sort of broader context. I'm considered both a subject matter expert and data custodian. [DC02].” |
|  | Capability and Confidence | Training | “We have data custodian guidelines and we're developing some checklists as well as some training for some specific data sets, mainly for the clearance and release processes of data. A big part of most people's role is …. to ensure that the data can be released and that there's no risk of identification or attribute disclosure. So that's probably the most difficult part of the role. There are training models and modules for that. We also have a data custodian forum where we can discuss all these issues and that kind of thing. So I do feel like there is a lot of support related to our responsibilities and how best to carry out our role. [DC02].”  “I’ve had to go down the path of getting the policies and procedures and I'm thinking that's not enough. I've gone back to the original legislation and done all that training as well, but I guess that's what my role is. It’s a bit of a backwards thing [DC02].  “I can't think of a particular training course that I participated in that instilled or gave me the skill set, but I think I've built it up over the course of my career. [DC04]”  “I wouldn't say that I've gone out and found a course that's taught me everything I need to know. To be quite frank, sometimes the learning has been through experiences. Fortunately, the processes that we have in place have been able to catch that. [DC06]” |
|  | Capability and Confidence | Confidence in the role | “I’m very confident and I feel capable to take that onboard, whether it's myself or delegated to the team. That we can do quite quickly and easily. What is more complex are sub-projects that are nested within the study. They have quite convoluted data extracts. [DC01]”  “I’ve used a lot of data sets and I've worked with the linked data sources, and I've also done some public reporting on the performance of the health system using multiple sources of linked data. I also have a fabulous team with great experience… We also have a secure place for data to be used. I'm very lucky I have a lot of infrastructure around me and I do have experience. [DC05]” |
|  | Role and Responsibilities | Role support | “It comes down as well to whether it's identified or identifiable data. If it's summary statistics on the number of participants who have smoked in the study, something like that is easy to ascertain and summarize and give out. But when you have individual-level data on 600 participants with specific questions, that's not something that I can just sign off on myself. It's a team effort and takes process. [DC01]”  “Each of us individually brings strength to the governance framework and enables us to draw off a multidisciplinary team with a range of skill sets depending on the scenario. We have deliberately established an independent external data governance committee who broadly, oversee what we do to ensure that what we're doing is absolutely right and meets with the expectations of the people whose data that we've got. [DC07]”  “That is the biggest strength of what we do because there's a recognition toward the complexity of being a data custodian and that there are all these different aspects to consider. The fact that we work collaboratively is also unique in itself because the expertise is typically siloed in terms of the technical teams, linkage experts, privacy and governance processes and stakeholder partnership relationship managers and many more. It's very rare that they all come together and communicate in the way that we do…..I would certainly say that from my experience - an organisation that has a data governance function should certainly have multiple people employed with various levels of expertise on a full-time, if not, part-time basis so that they are tasked with having dedicated roles because in recognition of the fact that there are all different levels of expertise that come together. [DC08]”  “I do quite often refer or differ to other areas [teams] for example. I would refer a lot of questions to a speciality area specifically because there's a lot of detail and I’d want to send those off for confirmation…. there are broader resources that I can draw on, but within my team, there are about 6. If I've got any concerns about providing access to data or the release of data, then I can go to our ethics, privacy and legal unit just to confirm that my understanding of the ethics coverage for the data collection is correct. While the final decision would be up to me as the custodian, I've got quite a pool of resources that I can draw on to make decisions if needed. [DC02]” |
|  | Organisational Considerations | Grant Funded | “We're not government. We can do what we need to do... a lot of the times it comes down to money and time and agreements and the contracts." [DC01] |
|  |  | Government Funded | “Part of the puzzle for us is actually how we manage the data and how we transform the data for it to be used,… we have many individuals who contribute to our governance process…we also have a lawyer on staff who is our privacy officer…we have a full-time sponsor and then we have another number of individuals who all have roles and responsibilities to play to ensuring that the data are well governed. Right from receipt of the data, through to disposal of the data through to management of practices who are contributing to the data. [DC07]”  “I have less than less than 10 within my specific team, but I have the option to refer or differ to other areas. I would refer a lot of questions to those area specifically because there's a lot of detail and you want to send those off for confirmation. If I've got any concerns about providing access to data or the release of data, then I can go to our ethics, privacy and legal unit to confirm that my understanding of the ethics coverage for the data collection is correct. If anything unusual comes up we've got statistical support that we can go to if there are any concerns or questions about specific suppressions or risk of attribute disclosure and that kind of thing. We can go and confirm some of those technical details and then, there's the analyst teams that have specific expertise in other specific data collections, if needed. [DC02]” |
| Barriers | Policy and Compliance | Legislation | “I would certainly draw similarities between the tertiary sector with the primary care sector in terms of opinions and attitudes, however, health and medical research are different in the way that the healthcare system from a State perspective is structured is different. …So there are different rules to follow, some of which you know can be a bit more lapsed, but then others can be a bit more rigorous…..That in itself is a significant body of work to continue to maintain, especially in recognition that standards change, legislation changes. You need to be able to be across those changes and embed them into the way the program is set up to make sure that it is meeting the required standards. [DC08]”  “The issue here is that our legislation around our data is at a State and Territory level so we have to contemplate provisions in the Health Administration Act, the Health Records and Information Privacy Act, Personal Privacy and Information Protection Act (PPIPA), and possibly in Public Health Act for certain data collections. All these have some sort of consideration when it comes to a decision around whether we release our data or not. Further to that our Information and Privacy Commissioner also has some statutory guidelines they're allowed to issue under the various referent PPIPA provisions, so there are statutory guidelines on research which we sometimes refer to, as statutory guidelines on the management of health services. We’ve also got policy directives set at a State level as well that influence the decision-making process. Further to that, we've also got some reforms to try and introduce more five safes framework type thinking as well. [DC06]”  “A lot of it is to do with the variations in legislation across States and territories, particularly in relation to health data. That is a really big one and then there is the sharing of data like Commonwealth data with States and territories and other organisations …. we sort of sit in the middle. [DC02]”  “The biggest barrier would be the legislative barrier that exists at the moment. The legislation currently prohibits [our specific] dataset from being shared, which is a particular barrier at the moment for us in releasing any of the data for analysis by researchers. [DC04]” |
|  | Data Handling Processes | Requests | “There's something on the website that articulates the process for how to apply for the data. When we have requestors send through requests for our linked data and related questions, we've got some fairly stock standard answers that we can provide them in terms of the pathways and rough expected times and some of the assumptions are going to those timeframes as well. [DC06]”  “Part of our transparency is to make sure that every utility of the data is visible, transparent and it's on our website. [DC07]”  “For each data collection there are tears of requests because they do need to be prioritised in a number of cases. For example, we could prioritize requests that come from a data provider to receive their own data, so insights back to Government, if it's, you know, to do with policy or planning those kind of requests can be fast tracked. Then if anything is related to an emergency situation, COVID for example was an excellent example of where some of the standard governance processes were shortcut in terms of the way requests were managed (more efficiently) rather than having to filter down through a number of processes. [DC02]”  “I've personally worked off best practice protocol where you just try and minimize the amount of data that's required and that's just from my days working in data linkage. I'm not sure if I've actually ever been shown or directed towards any kind of policy. For [our program] all we have done is de-identify to the subproject level so that when the data goes out they don't receive identifiers, they would just receive numbers from one through to 500 instead….But doing all of that takes time. Making sure that you've done it correctly and validating everything just adds to the time required to release the data. Sometimes I think if we had more guidance on exactly what we can and can't do, that would be very useful…..And that's probably where a lot of the confusion can come from sometimes from not having these clear guidelines. Even down to how you share the data, because you know you try and do the right thing, but even how I'm allowed to share that [data] between somebody who's here in WA, or someone who's in New South Wales I don't have clear guidelines on how it should be done. [DC01]”  “We treat every single application as an individual request and evaluate it on its merit and scope. Since the data governance committee only meet every two months, we do have scope to fast-track something through data governance out of session if we need to. However, my anecdotal observation is everybody wants the data yesterday and very few people really respect the multiple steps and hoops in the process and the length of time these things actually take. If you're not careful, you would fast-track everything because everybody thinks it's urgent and it's an emergency, and it all has to be done yesterday. And very few of them really fall into that bucket. [DC07]”  “I mean our goal is to make everything as expedient for researchers as possible. That's sort of our strategic goal. A minister can't do anything special because the request still has to go through an Ethics Committee, so they have to do their ethics first. It doesn't matter who they are [DC05].”  “One of our projects was developed to provide analytical support and evaluation for a particular program which required various levels of consent across the 3 or 4 forms. For that one program, I found it to be both very confusing and difficult. [DC04]”  “It's [ethics] completely up to the applicant. If you're putting together a [funding] application, for example, you're probably not going to do ethics until you know you've got the funding, and with those, their application might come in because they want to put their application to [the funding body] with a positive data governance approval. [DC07]” |
|  | Data Handling Processes | Request Assessment: Custodian experience | “People aren’t always aware of the complexities of the data and associated workflow….It's not perfectly curated and it's not nicely entered. It comes in a ‘warts-and-all’ approach and if you don't understand the workflow that contributes to how the data manifests and you can't articulate those characteristics and limitations and you're going to do a really bad job of actually unpacking the insights and the intelligence. [DC07]”  “This is one of the challenges that exist with data custodianship because you do want to see data being used, but you also want to see your data being used in a safe and responsible manner. I really want to see that there's a public good that can arise from the use of that information. Sometimes assessing that public good takes either a long time or to see that the application can suitably demonstrate that it is in the public interest, requires multiple iterations. [DC06]”  ”You have to have the lens of the author of the data when you're considering the data utility. If you don't consider the needs, wants, and drivers of the source and understand the perspective they'll be coming from when they're making decisions, then you're not fit to be a data custodian. You have to understand the lens of both them [GPs] and the patient in trying to help navigate those decisions. Those skill sets are pretty rare too. [DC08]”  “I think there would be some benefit, from having a custodian sit in on some of these Ethics Committee meetings just to see what the process is like and how thorough people are. If a project is reasonably well described, the circumstances are understood and there aren’t any issues, projects should be able to be reviewed fairly quickly. [DC06]” |
|  | Data Handling Processes | Processing time | “Requests can take anywhere from three weeks to be fully processed and cleared up to 6 to 8 months just depending on the complexity of the data, and particularly how many layers of governance it needs to go through. I would say eight months is probably the longest. That I've been involved with. [DC02]”  “I’m proud to say that on average it's taken from start to finish three months, however that excludes datasets from the State Health Department because I would say that they would be considered to be a barrier. I think they are aware of it and working on it, following the recommendations of [a recent review] into the capability of data linkage. They're finally making those changes. So I'm hopeful. [DC04]”  “If everything were to go to plan, and if the application were timely around about the time of data governance, you probably be looking at around about 8 weeks and it can be anything through to several years. [DC07]”  “For linked datasets that are subject to HREC [Human Research and Ethics Committee] approvals I try to make sure that I've provided at least first-pass feedback or approval if it's clean within two to four weeks. …. For the aggregate requests, because there's a lot more work involved there, there can be a bit of back and forth in terms of refinement and requests to related data which can take about four to six weeks sometimes. [DC06]” |
|  | Data Handling Processes | Request complexity | “Part of the reason is that the more data custodians that are involved, the longer it takes because obviously each one is busy and each one has to wait for the other as well. Then it has to go to ethics and they don't meet necessarily every month. Some do, some don't, and so getting that first approval, even if you have to do amendments afterwards, is very difficult. That's just with plain Data Custodians. That's not even including researchers or committees, it's not even adding, say, Aboriginal Health and Medical Research Ethics Groups which adds another layer of complexity. [DC05]”  “Every time a new request comes in we have to ask ourselves: ‘How's it going to go this time?’; ‘That's ever so slightly different….that wording is a little bit different. What does that actually mean in this context? [DC02]”  “There is not enough specificity in the research questions that are being posed to provide data custodians with the level of comfort to reassure them that the data is being used for an appropriate purpose. [DC04]”  “For example, if you need condition coding for a cohort that we haven't coded before, you can add a period of time to that because there's some extra work to be done before the data can be prepared. [DC07]” |
|  | Data Handling Processes | Threat to research | “Part of what we're working with takes researchers a long time and many early and mid-career researchers will give up. It is really arduous going through the different data custodians - the back and forward to ethics…. It’s not ideal the way it is currently. It's time-consuming, and expensive, which means that a lot of people never get their data. They give up even if they've got the resilience to keep going. [DC05].”  “So the barriers as I see them are the capacity of the relevant data custodians to be able to make decisions as well as taking the role extremely seriously and being very cautious around their agreement and approval process. We have one dataset from the State Health Department that is taking over 2 years…It feels like we’re in a perpetual state of requesting the same data. I’ve lost track of how many new request forms we’ve had to fill in for the same data. [DC04].” |
|  | Data Handling Processes | Capacity | “I understand the importance and seriousness of this role and the gravity with which some of the data custodians take this on, however, it does sometimes make it difficult for me to achieve my role in providing or granting access to researchers or analysts within government, because of the fact that in some agencies there is only the one data custodian and they are the only ones who can give me the green light as to whether or not a project is able to access their data [DC04]”  “Some requests can be a little bit complicated. We may need to have a few iterative meetings with the requesters for clarification, and I just don’t have the time for that. Most of that kind of stuff could be delegated to the person who's doing the analysis. [DC06]”  “I don't know what it’s like in other [Australian] States, but in [our State] there's been a massive loss of those experienced researchers due to COVID with a lot of positions being let go and as a result [the State] has lost some really high-level researchers. There's going to be this real gap there for mentoring and anything we can do to support that capability development would be important. [DC05]”  ”We’ve got to get started by supporting Australian researchers in this space. It's not that only early career researchers can use our [platform] - anyone can apply to use it. It will certainly help meet our goal and related strategies to support that development in Australia by using these data sources. [DC05]” |
|  | Policy and Compliance | Consent | “Receiving data where consent is required or legislated can be a problem. Some datasets like the MBS and PBS are challenging because people don't technically give their consent for the data to be used for other purposes. That makes it really complicated for how and what it can be used for, and who can access it. [DC02]”  “There could be a lead body within the general practice community that can support a framework for consent to collect information universally across the sector from patients and have it used for specified purposes. I think there are opportunities there. [DC08]”  ”One thing that could help is developing good consenting material. Rather than explain to participants, what data linkage is, it’s an opportunity to tell them what it's about and what it does - without all the words legislation which was all included in it. [DC05]”  “Whilst you've got the Privacy Act and standards around non-identifiable data, there's a specific framework and code for the indigenous population around how they view the secondary use of their information. Any good data custodian would need to look at that as well and take the time to consider not only the cultural differences for indigenous peoples but also, the linguistically diverse communities. [DC08]” |
|  | Organisational Practices | Organisational culture | “It does take a long time. Yeah, It takes years to develop and only seconds to break. [DC08]” |
|  | Organisational Practices | Management support and domain knowledge | “In my experience, holding onto the data and not preparing it for release has been more of an indicator of potentially not understanding what the actual risk is and how best to mitigate it. We do see that in for example primary care. [DC02]” |
|  | Governance and Authority | Political will | “Those in the echelons of leadership don’t understand what data science is and perhaps we're still educating upwards. I'm wondering if it's further up the chain and whether it’s associated with political will that’s a particular barrier to data sharing. I think there is that element of not even understanding how your data can improve things or inform better decision-making. [DC04]” |
|  | Organisational Practices | Questionable authority | “Should individual GPs have the responsibility to determine whether that is risky or not? Whether that breaches their client and patient privacy or not. I think that's too much [responsibility] for GPs or practice managers for example and I think that this may be outside of their scope of expertise. [DC02]”  “There’s also a lack of knowledge about responsibilities and interpretation of legislation. I mean GPs shouldn't be required to interpret the privacy legislation in order to release data. [DC02]”  “It shouldn't be one person deciding whether this does or doesn't happen. It's the same in general practice. However, they're moving from the historical way of a practice manager saying ‘yes, you can have our data’, to a clinical governance Committee now overseeing what happens to the data. [DC07]” |
|  | Organisational Practices | Risk aversion | “The question always comes up as – ‘Can we share this?’ and the easy answer is yes because it's de-identified data. It needs to be really clear … you can share this data because it's not under the Privacy Act and therefore you won't get sued over it. It's all about making people less risk-averse with sharing data because we want to, it's useful and it's important for health research. [DC03].”  “Even though all the people are de-identified, we still have quite strict guidelines about what can and can't be included, just so that organisations, providers or participants are not identified. [DC05]”  “We collect non-identifiable data from consumers, we don't have to inform them that we are collecting their data, but it is best practice to do so irrespective of the format because the Privacy Act does have a provision in there to say if an organisation is collecting non-identifiable data for secondary use, then consent is not required because it's not considered to be personal information. For some, that [understanding] would be taken in black and white, but our approach is to go beyond the standard and take the opportunity to build our relationship with data providers and notify them how their data is being used. [DC08]”  “The other murkiness in the market is the perception and differing views about what you can and can't do with de-identified data. It’s not consent. Deidentified data falls under a different bucket to data that is largely governed by the Privacy Act and I think that has been, and will continue to be a challenge while people try and understand the differences between using identified data -which does require consent; and deidentified data. [DC07]”  “What I found when talking to people in these data spaces is that they know that they should be doing these things, but it's not necessarily having the time to write them [policies/guidelines] or have the time to understand them - There's no checklist for a data custodian to say - these are the things that you should be doing on an annual basis. [DC03]” |
|  | Data Handling Processes | Dataset limitations | “In the case of the PIP QI, that data became so aggregate that it was almost impossible to do anything with it because it was almost meaningless. It's important to get that balance of utility and access. [DC02]” |
| Facilitators | Centralised Data Sharing Models | Infrastructure and workflow improvements | “It’s like getting that same service every time a request comes in. You know exactly what you're going to get and what you should expect rather than it being a new experience every single time. …..So having a consistent platform or framework and transparency of process and experience, as well, is a way of really generating that trust. I think that data is being well managed and that it's safe. [DC02]”  “If you're busy doing something else within the team, it can't be done. To me, there's a level of data architecture and engineering that has to occur within the project to streamline these data requests. We have invested in something called the …… data hub which is a system that the institute is developing to streamline these data requests. It's taking a while for us to have a system that is easy to use and works very well. [DC01]”  “We're wanting to set up support for early career researchers, and a process which means that the data will be ready and that they won't have to wait for all the complicated and variable request processes. Our plan is to develop a program where we can provide more support by creating opportunities and developing the capabilities of young Australian researchers. Linked data is hard work. It's big. It takes experience and great skill and we’ve got to get started by supporting people in this space. It would be closely aligned with better governance because then you've got more capable people working with the data and building that capability. [DC05]”  “I feel Australia needs is a centralised coding sharing platform, similar to a community of practice, where people interact with one another and share their lessons learned and any [analytical] code where they would be willing to share learn from…For example: Let's write your big SAS program to identify your cohort, your variables of interest, your outcome variables that will be included and let's code them and learn how to work with different datasets and link them together. [DC05]” |
|  | Centralised Data Sharing Models | Centralised consent | “Having centralised, dynamic consent and a way of having that consent brought across a number of data collections at practice level or provider level State and Territory levels, that kind of thing would be a dream come true. [DC02]”  “There are opportunities for peak bodies to play in the role of integrating consent, but then there are risks to that as there are benefits, and balancing them is the biggest challenge in the sector because that will enable these sorts of digital processes and strategies to happen. You've also got to look at harmonising the consent process, but also harmonising it in a way that is open and transparent. If consumers want to look at where their data is going and what it's being used for, that they're able to do that and the infrastructure behind something like that is colossal. [DC07]”  “Something that would help would be some level of informed consent, that is collected at the point of data collection. That way you are relying on the processes that you’ve developed to disclose that information. [DC04]” |
|  | Centralised Data Sharing Models | Common identifier | “I think having an MLK [master linkage key] in each State and having that aggregated in a safe, secure place that's a bit separate would be really good. [DC05]”  “A lack of access to the IHI [individual healthcare identifier], to be able to find the same person across the data set, is a challenge. [DC07]” |
|  | Exemplars of good practice | Exemplars of good practice | “I would say that NPS MedicineWise has the right idea around engaging with consumers and the way in which the program has been set up and the way all the different facets connect together. They seem to have a passion for engaging with consumers to close that gap that exists within the other sectors . [DC09]”  “We look to New South Wales and their LUMOS program and New Zealand as the prime examples or the aspirational jurisdictions, really. The fact that they are making outcomes-based decisions is something that I would love for our state to aspire to. [DC04]”  “Obviously the NHS but, their system is really quite different to ours. Their transparency, about the way they use data, is really good and that sort of centralized system for people being able to opt-out or opt-in and provide that consent…. We do like the work that's being done in WA. Big fans of that as well as like the Lumos project in New South Wales which you know they've really pushed a lot of those pre-existing barriers about linking general practice data. [DC02]”  “The work that Manitoba’s doing for example around accessibility has been amazing. Some of the Canadian organisations are really doing great things there. They've got advantages over us in some ways that some of their admin data is a little more useful from a primary care data perspective. [DC05]”  “When we think about how willing people are to share financial information to access their information using apps to, transfer funds and data between different people. It's largely because there's that assurance of security, and we've been looking a lot at - how is it that people are so willing to share information about their finances and their money and assume that it's going to turn up where it should and I think it is the financial sector that is seen as being highly secure, highly regulated and highly technical. We find that quite interesting. [DC02].”  “There's actually a big leap between people’s willingness to share anything on social media and subscribe to anything if it gets them to a particular platform, to sharing their data for the purpose of allowing public agencies to do something better. I feel like there's this massive gap that we need to bridge to build that confidence. [DC04]” |
| Future State | Exemplars of good practice | What does success look like? | “I think a successful governance process is one that almost isn't seen because it's exposed. It's consistent, it's standardised, it's known and it's trusted. [DC02]”  “I think success would be an open and transparent process for data users to access the data in an appropriate and timely manner, for the purpose of delivering public value. [DC04]”  “Ideally, these projects would be coming through where people have had adequate time to consider and think things through the privacy principles and the data security issues. That would make the decision to approve or not approve a lot easier. I think it's just a case of providing additional guidance on what to ask for more specifically. [DC06]”  “There’s a huge opportunity to connect, certainly primary and acute care pathways together, and the benefit that will have not only for the research community but for consumers and health practitioners that work across those fields. We’d have such an amazing and rich data source that can really help drive policy changes in various areas. Certainly, reducing the siloed duplication that's out there would be a huge benefit. [DC08]” |
