# Supplementary material for: Attitudes towards data access and sharing health data for research: a case study of Australian data custodian perspectives
Source: Health Inf Manag. 2025 May 3;55(2):315–27. doi: 10.1177/18333583251329533 (PMC13187238; doi:10.1177/18333583251329533)
Supplement: sj-docx-2-him-10.1177_18333583251329533 – Supplemental material for Attitudes towards data access and sharing health data for research: a case study of Australian data custodian perspectives [file sj-docx-2-him-10.1177_18333583251329533.docx]

| Theme | |
| --- | --- |
| **Understanding of the role** | |
| **Question** | **Probe** |
| What is your understanding of what a Data Custodian’s role is? | - What are your responsibilities? - Do you feel you have the confidence and appropriate training for this role? - What level of authority do you have currently or would like to have in the future? |
| How long have you been in the role? | - Identify their title - Ascertain how they got into the role.   - Promoted from within (skills developed over time?)   - Already had the skills and training and came from an external organisation - How many datasets are you responsible for? - Is there redundancy built into data custodianship? |
| Do you have a strategy mentor? | - Does your organisation have a digital / data strategy? - Who would you model your data strategy after? |
| **Barriers to the role** | |
| What are some of the barriers that prevent you doing your role | - On average how long does it take for a data request to be processed? - Is there an established governance and approval process?   - Is it transparent to the requestor? - Is it possible to fast-track requests depending on the priority (i.e., ministerial, or internal request)? - Potential probes: - Company - Cultural (i.e., sharing requirements come from the top?) - Legislation and regulation impediments? - Consumer - Privacy, security - Competence managing risk - International - Uncertain of potential vulnerabilities from foreign cyber actors |
| **Opportunities for improvement** | |
| What does data success look like to you? | - Identify familiarity with frameworks - Is there appetite in learning from others?   How would you improve the current system |
| In your opinion, how do we get people to share their data? | - Cultural (i.e., sharing requirements come from the top?) - Legislation and regulation impediments?   Data security? |
